# Representational maps in the brain: concepts, approaches, and applications

**DOI:** 10.3389/fncel.2024.1366200

**Published:** 2024-03-22

**Authors:** Takahiro Noda, Dominik F. Aschauer, Anna R. Chambers, Johannes P.-H. Seiler, Simon Rumpel

**Affiliations:** ^1^Institute of Physiology, Focus Program Translational Neurosciences, University Medical Center, Johannes Gutenberg University-Mainz, Mainz, Germany; ^2^Department of Otolaryngology – Head and Neck Surgery, Harvard Medical School, Boston, MA, United States; ^3^Eaton Peabody Laboratories, Massachusetts Eye and Ear Infirmary, Boston, MA, United States

**Keywords:** sensory coding, large-scale recording, neuronal population activity, neuronal tuning, brain topography

## Abstract

Neural systems have evolved to process sensory stimuli in a way that allows for efficient and adaptive behavior in a complex environment. Recent technological advances enable us to investigate sensory processing in animal models by simultaneously recording the activity of large populations of neurons with single-cell resolution, yielding high-dimensional datasets. In this review, we discuss concepts and approaches for assessing the population-level representation of sensory stimuli in the form of a representational map. In such a map, not only are the identities of stimuli distinctly represented, but their relational similarity is also mapped onto the space of neuronal activity. We highlight example studies in which the structure of representational maps in the brain are estimated from recordings in humans as well as animals and compare their methodological approaches. Finally, we integrate these aspects and provide an outlook for how the concept of representational maps could be applied to various fields in basic and clinical neuroscience.

## What is a representational map?

### From single cells to maps

Electrophysiological recordings of single neurons have been extremely influential in our understanding of how neural activity is modulated by sensory stimuli and their physical properties, such as pure tone frequency or drifting grating orientation (Galambos and Davis, 1943; Hubel and Wiesel, 1962). However, compared to the sensory epithelium and early processing stations in the brainstem, neurons in higher-order areas in the cortex reconstruct stimulus identity from an increasingly cognitive, rather than purely physical, point of view. Single neuron representations become more selective to complex features (Bizley et al., 2007; Bizley and Walker, 2009; Sieben et al., 2013), and tolerant to identity-preserving variations in scale, position or intensity (Rust and DiCarlo, 2012). Further, sensory responses in higher order areas can be influenced by learning and behavioral states (McGinley et al., 2015; Vinck et al., 2015). While these findings were traditionally based on recordings of limited numbers of single neurons, a qualitative change in analyzing sensory responses was introduced through the emergence of techniques to measure the activity of large neuronal populations, particularly in the cortex (Saxena and Cunningham, 2019; Steinmetz et al., 2021; Grienberger et al., 2022). This technical development has created the possibility of new modes of interpretation, beyond the single-neuron tuning curve, to capture the self-organizing dynamics that underlie the representations of sensory input at the population level (Brette, 2018; Ebitz and Hayden, 2021; Panzeri et al., 2022). The analysis of population dynamics enables us to understand how the brain maps relevant information onto an internalized space to inform perception and behavior.

### Representational maps in the brain

To navigate efficiently through the external world with its ever-changing demands and challenges, our brains continuously store and process information. This information is represented in neural activity that informs and underlies behaviorally relevant computations (Marr, 1982). The structure of these internal neural representations reflects the relationships between their real-world counterparts, optimizing the inferences drawn from them (Buckner and Carroll, 2007; Behrens et al., 2018; Barron et al., 2020; Park et al., 2020).

In this review, we focus on the structured representation of informational entities in the brain, or the brain’s *representational map,* as a fundamental mode of organization across different cognitive domains (Behrens et al., 2018; Kriegeskorte and Wei, 2021). Although the term ‘map’ typically refers to a two-dimensional representation of relationships in physical space, it is used here in a much broader sense (Brette, 2018). In a traditional topographic map of a town, for example, the physical distance of items like buildings and streets corresponds to their mapped distance. In specific cases, some properties of the neural representational map are similarly mapped in physical space, e.g., the cortical surface, leading to topographically organized sensory representations such as auditory tonotopy or retinotopic organization in visual cortex (Dräger, 1975; Guo et al., 2012). However, in a more general sense, neural representational maps depict cognitive relationships as similarities of population activity in an abstracted neural activity space (Shepard and Chipman, 1970; Edelman, 1998; Kriegeskorte et al., 2008a). Thus, a high similarity in activity patterns would correspond to a close relationship between two items (Connolly et al., 2012). Importantly, the intentionally loose term “*relationship”* can describe various properties, allowing the simultaneous generation of multiple, complementary representational maps in the brain.

For our review, we define the concept of a representational map in the brain as follows:

1. A representational map exists in a space that is defined by neuronal activity.
2. Relevant representational entities must be distinguishable from each other in this map, i.e., associated with distinct activity patterns.
3. The map depicts relationships between representational entities in a way that informs neural computations.
4. These relationships are encoded as similarity or dissimilarity of activity patterns.

The useful properties of a representational map are clear when contrasted with hypothetical patterns of brain activity in which each representational entity is associated with a unique activity pattern that is equally distinct from all other entities. Despite its efficient coding, such a representation would be limited in its ability to guide decisions and behavior (Brette, 2018). In contrast to a map, this scenario is comparable to a randomized list of items, which provides no information on their relational structure. A representational map, however, not only enables the identification of representational entities and their relationships to each other, but also enables inferences about novel stimuli (Behrens et al., 2018; Pashkovski et al., 2020), since the activity pattern evoked by a novel stimulus will correspond to a particular position on the representational map, providing implicit relational information to other stimuli.

As representational maps exist in an abstract space defined by the activity of the neuronal population, the dimensionality of this space is – in principle – as large as the number of neurons in that population. As the number of neurons accessing sensory input typically increases from the sensory epithelium to higher cortical areas, the dimensionality of representation, in theory, could grow massively. In practice, however, the relevant neural activity space is constrained by many variables including metabolic, biophysical, and anatomical factors, as well as by the constraints of real-world contexts and associations. Many neurons in a recorded population have highly correlated activity (Pillow et al., 2008; van den Brink et al., 2019; Gava et al., 2021), or contribute relatively few spikes to the overall activity during the investigated event or time period (Willmore et al., 2011). Representational maps thus tend to occupy a lower-dimensional topological subspace (Santhanam et al., 2009; Sadtler et al., 2014; Luczak et al., 2015). The structure of neuronal activity within a high-dimensional space is often described by manifolds (Jazayeri and Ostojic, 2021), which relate to the concept of a representational map (Gallego et al., 2017; Mitchell-Heggs et al., 2023). However, a representational map puts particular emphasis on the relational structure of the represented entities. As such, a manifold can be rather used to describe the general geometry a representational map occupies in neuronal activity space, where the structure of the map itself highlights the relational information of the represented items.

### Representational maps of sensory features: topography and beyond

As mentioned above, classical examples of low dimensional representational maps – namely, sensory topographic maps where functional properties follow an anatomical organization – can be constructed from the activity of neurons whose feature tuning varies according to their positions on the two-dimensional cortical surface. Reflecting the organization of projections from the sensory epithelium, topographically organized maps of stimulus features are a hallmark of sensory systems (Dräger, 1975; Merzenich et al., 1975; Kaas, 1997; Li et al., 2023). In the primary visual cortex (V1), neurons are arranged in a topographic manner that corresponds to the position of stimuli in the visual field (Allman and Kaas, 1971). V1 neurons respond to stimuli in a specific location, with adjacent neurons responding to adjacent locations in the visual field. Studies in the somatosensory cortex (S1) have similarly demonstrated representational maps for different sensory areas of the body (Nelson et al., 1980). In the auditory cortex, local neural tuning to pure tone frequency progresses systematically along the cortical surface (Merzenich et al., 1975). Classical sensory cortical topography fulfills the representational map criteria laid out previously: pure tone frequency is mapped onto a low-dimensional space, where the distance between frequency regions corresponds to the similarity of the stimuli, albeit the degree of organization can vary across species (Kaschube, 2014). Furthermore, a topographical organization of functional dimensions, that reach beyond simple physical properties have been described in the language processing system (Huth et al., 2012, 2016).

However, topographically mapped sensory properties are not the only features critical for identifying and analyzing relevant stimuli. There are complex features that researchers were unable to find a topographical organizing principle. For example, it is known that auditory cortical neurons are sensitive to diverse stimulus properties, from physical variables such as intensity, to perceptual variables such as pitch (Bendor and Wang, 2005). Most of these features are not inherited in a topographically organized ‘labeled line’ structure from peripheral projections. Further, some relevant stimulus attributes, particularly those that are complex and multidimensional, may only be represented distinctly at higher levels of the processing chain.

The emergence of higher-order perceptual representations may partly be due to an increasing convergence in network connectivity (Singer, 2021). Convergence enables two processes that aid the formation of cognitive categories and the ability to judge perceptual similarity beyond a simple analysis of physical attributes: First, each individual neuron in a cortical area has access to more of the sensory environment than lower-level neurons due to the expansion of the number and diversity of inputs (Felleman and Van Essen, 1991; Guillery and Sherman, 2002; Babadi and Sompolinsky, 2014). Second, network activity encoding low-dimensional stimulus relationships (e.g., intensity or frequency differences) can be broadcast to a large population, generating diverse combinatorial codes, and distributing information about these relationships over a higher-dimensional space (Fusi et al., 2016; Rossi-Pool et al., 2021). This higher-dimensional structure, with both hierarchical and recurrent network elements, is remarkably robust at generating useful cognitive constructs that allow for the analysis and interpretation of external events, whether familiar or novel, despite the fact that only few feature dimensions map on the cortical surface. With this, the concept of a representational map goes beyond classical topographically organized maps. Hence, topographical maps can be understood as a subset of the more general definition of representational maps in the space of neuronal activity, as described above.

Sensory items are represented on multiple representational maps across a processing hierarchy simultaneously. The structure of these maps will differ, however. Here, analogous to single cell receptive fields, representations of physical properties will be increasingly replaced by perceptual properties and the similarity of activity patterns will rather reflect perceptual similarity (Op de Beeck et al., 2008; Carlson et al., 2014). Importantly, this can lead to the phenomenon that stimuli that are represented distinctly on low-level representational maps, could be mapped together on high-level representational maps, reflecting the formation of perceptual categories (Connolly et al., 2012).

Representational maps can also be found at further stages of a sensory-motor transformation, such as the hippocampus (Park et al., 2020; Nieh et al., 2021), cortical association (Carota et al., 2017; Whittington et al., 2022; Nelli et al., 2023) and motor areas (Santhanam et al., 2009; Churchland et al., 2012; Gallego et al., 2017; Keller and Mrsic-Flogel, 2018). Multiple activity patterns each confined to lower processing complexity, could be interlinked with each other in larger circuits to form higher-order, potentially multisensory and complex representational maps (Popham et al., 2021). Moreover, such parallel representational maps interconnected by feedforward, but also feedback connections can help to interpret mixed neuronal activity patterns in response to similar stimuli in changing context (Rigotti et al., 2013; Deniz et al., 2023; Kira et al., 2023): Based on the specific context, a certain stimulus might be represented on different representational maps, which are selected to optimize behavioral inferences drawn from the map in this situation. Thus, the neuronal representation of stimuli by mixed-selectivity neurons allows their embedding on different representational maps and therefore enables flexible interpretations based on different contextual information.

Although the representational map approach offers a unified perspective to extract relevant features of neuronal activity at both high and low levels of the processing chain, in this review we will focus on sensory representational maps.

**Figure 1:**
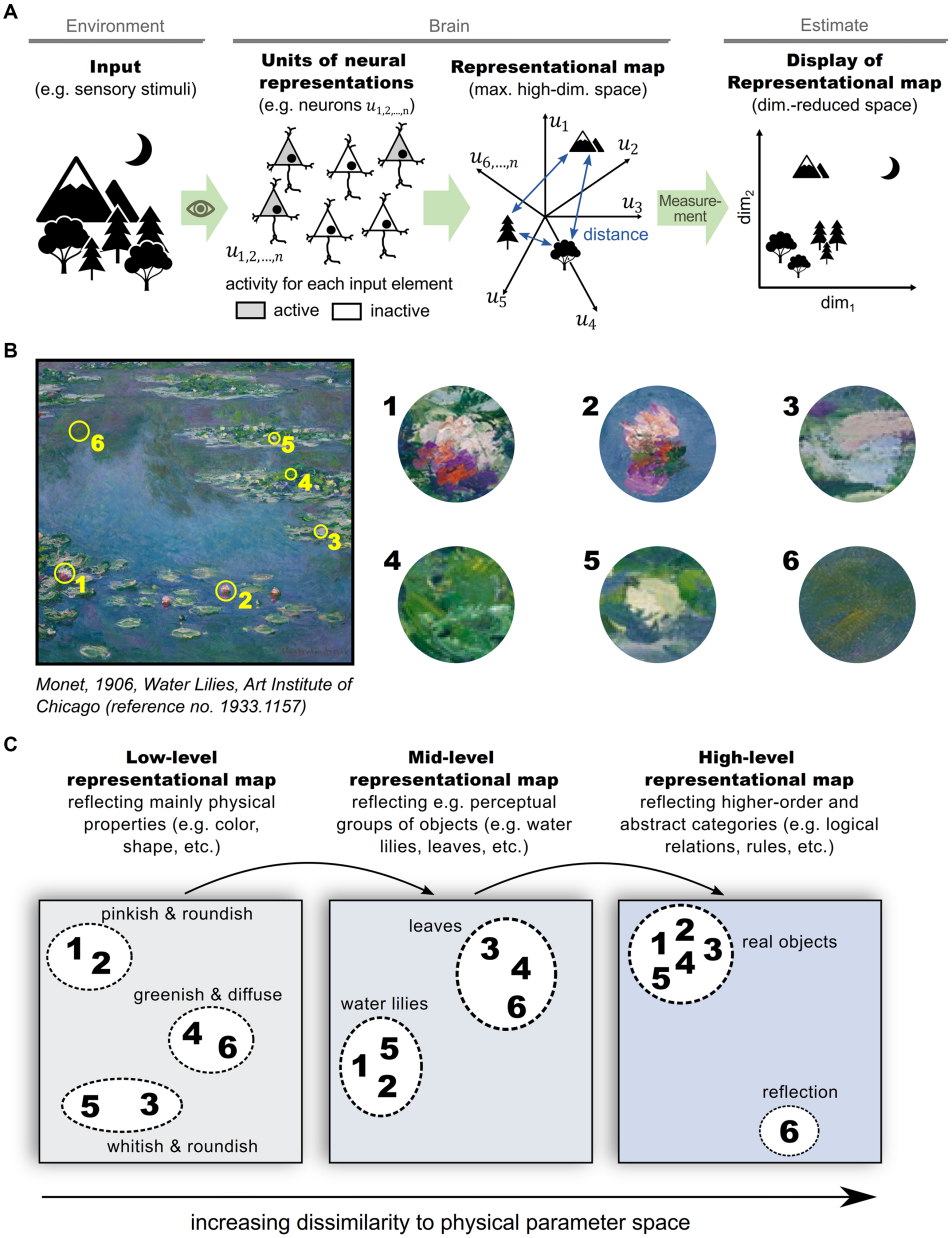
Representational maps in the brain. **(A)** Schematic illustrating the formation of a representational map in the brain and the experimental estimation of its structure: A set of informational entities, e.g., different physical stimuli, are perceived by sensory organs and transformed into neural representations in a high-dimensional space of neuronal activities in the brain. The structure of the representational map can be estimated by neural measurements (e.g., fMRI, electrophysiological recordings, calcium imaging, etc.) and are typically displayed and interpreted after a reduction of dimensionality. **(B)** Exemplary sensory input scene that comprises various informational entities, such as water, water lilies and leaves. **(C)** Illustration of multiple, complementing representational maps in the brain. In primary sensory regions, representational entities are predominantly mapped according to their physical properties like color, shape and orientation (left panel). In hierarchically higher brain areas, representational entities organize according to perceptional categories (mid panel), or even abstract categories (right panel).

To experimentally estimate the structure of a representational map in the brain, a sufficiently large and representative number of neurons need to be sampled and analyzed (Shepard, 1980). Although the relevant structure can be captured in fewer dimensions than the number of recorded neurons, it is typically larger than two or three (Haxby et al., 2011; Stringer et al., 2019), complicating visualization. To overcome this problem, two or three dimensions that explain the most variability in neuronal activity are often selected for visualization using one of various dimensionality reduction techniques (Yu et al., 2009; Ganguli and Sompolinsky, 2012; Cunningham and Yu, 2014). Figure 1 schematizes a representational map of a sensory scene in the brain and the experimental estimation of its structure in a dimension-reduced form (Figure 1A). An equivalent process can be applied to various hierarchical levels of information processing in the brain (Figures 1B,C), ranging from basic sensory stimulus representations in a perceptual context (Figure 1C, left), to abstract representations of environments and cognitive schemes (Figure 1C, right) (Tolman, 1948; O'Keefe and Nadel, 1978; Schuck et al., 2016; Rubin et al., 2019; Park et al., 2020). In this schematic, feedforward sensory processing across hierarchical brain structures is emphasized, but it should be noted that feedback from higher-order brain structures likely impacts a representational map. Importantly, multiple representational maps at different processing levels are not exclusive, but rather complement each other (Jacobs and Schenk, 2003), and should be read out simultaneously in order to optimize behavior (Semedo et al., 2019).

In the following sections, we first highlight examples of how representational maps can be estimated from neural activity across different model systems, including human studies. We provide a conceptual template for estimating representational maps from neuronal population data in a given model organism, and consider the conditions under which a representational map can arise. Finally, we discuss the benefits and limitations of the representational map approach and present an outlook for how it can be applied to clinical neuroscience.

## Example studies from the literature

### Representational maps in primates including humans

A widely used approach in the cognitive sciences to estimate the structure of a representational map is a Representational Similarity Analysis (RSA, Kriegeskorte et al., 2008a). RSA is grounded in psychological studies to match similarities among input (e.g., stimulus) properties and similarities among the internal representations (Shepard and Chipman, 1970) as well as the analysis of single-unit recordings using population vectors (Georgopoulos et al., 1986; Dimsdale-Zucker and Ranganath, 2018). RSA was specifically formulated by Edelman (1998) and first applied to human fMRI data (Edelman et al., 1998). RSA is a technique that positions representative entities relative to each other by computing a two-dimensional matrix, where each element corresponds to a dissimilarity score for a given pair of vectors describing the population activity evoked by the presentation of a given sensory stimulus (Kriegeskorte et al., 2008a; Kriegeskorte and Kievit, 2013).

**Figure 2:**
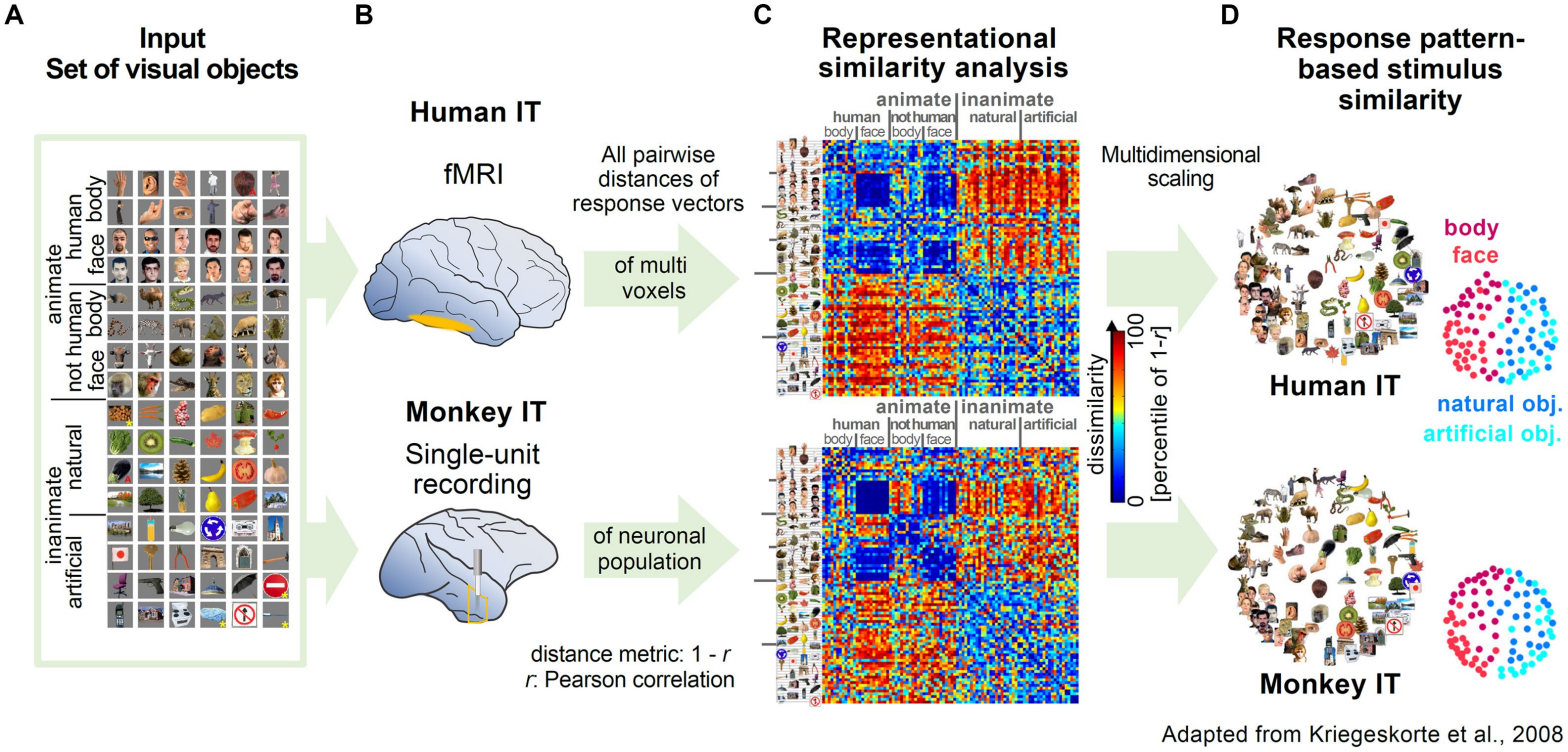
Examples of representational maps in the human and primate brain for a common set of visual stimuli. **(A)** The image set from different categories presented to monkeys and humans. Reproduced, with permission, from Kriegeskorte et al. (2008b). **(B)** Measurement of brain activity in human and monkey inferior temporal cortex (IT). In human IT, neuronal activity was measured with high-resolution blood oxygen-level-dependent fMRI. In monkey IT, single unit activity was recorded extracellularly with tungsten electrodes. **(C)** Representational Dissimilarity Matrices (RDM) for monkey and human IT. The dissimilarity of the two response patterns to a given pair of stimuli was calculated and displayed as color-coded RDM. The Dissimilarity measure was computed as 1 – r (Pearson correlation). **(D)** Stimulus arrangements reflecting response pattern similarity in IT for monkey and human. Multidimensional scaling was applied to reduce the dimensionality of the RDM from C. In the resulting representational map, images close to each other evoked similar response patterns.

The influential work by Kriegeskorte and coworkers highlighted the versatility and robustness of RSA by applying the methodology to two different data types (fMRI and single-unit recording) from two different species, human and monkey. Experimenters presented subjects with a wide range of images of animate and inanimate objects while measuring activity in the inferior temporal cortex (Figures 2A,B; Kiani et al., 2007; Kriegeskorte et al., 2008b). Representational maps were estimated by analyzing representational dissimilarity matrices (RDM) constructed from multi-voxel and population activity patterns (Figure 2C). Interestingly, the estimated representational maps showed a clear structure reflecting a categorical grouping of objects according to various features. Furthermore, they observed a striking similarity in the organization of the representational maps, despite independent data acquisition with different recording methods (Figures 2C,D). Most importantly, the high correspondence in structure of the maps across different species, suggested that the semantic mapping of the various visual objects shared a large degree of similarity.

Up to date, analysis of representational maps has been used in the form of RSA for neural recordings from different experimental techniques such as EEG/MEG (Su et al., 2012; Cichy et al., 2014; Kaneshiro et al., 2015; Wang et al., 2020) and PET (Kao et al., 2021), covering different sensory modalities like vision (Cohen et al., 2014; Kaneshiro et al., 2015; Cichy et al., 2016; Guntupalli et al., 2016; Wardle et al., 2016; Groen et al., 2018; Xu and Vaziri-Pashkam, 2021; Luo and Collins, 2023), audition (Perez-Bellido et al., 2018; Berezutskaya et al., 2020; Mattioni et al., 2020; Bodin et al., 2021), somatosensation (Lee Masson et al., 2018; Liu et al., 2021; Ariani et al., 2022; Kryklywy et al., 2023), olfaction (Fournel et al., 2016; Iravani et al., 2021; Kato et al., 2022) and motor planning (Ariani et al., 2022).

### Representational maps in rodents

In contrast to most human studies, where neuronal activity is typically recorded with limited spatial resolution using functional magnetic resonance imaging, animal studies allow recordings of neuronal activity with single-cell resolution. In the last decade, through technological advances in genetics, *in vivo* microscopy, and electrophysiological methods, large datasets of neuronal activity have been acquired, particularly in rodent models. In these experimental settings, the utility of traditional single-cell tuning curves starts to diminish and the need for less biased methods to describe the complex structure of the neuronal population data emerges.

**Figure 3:**
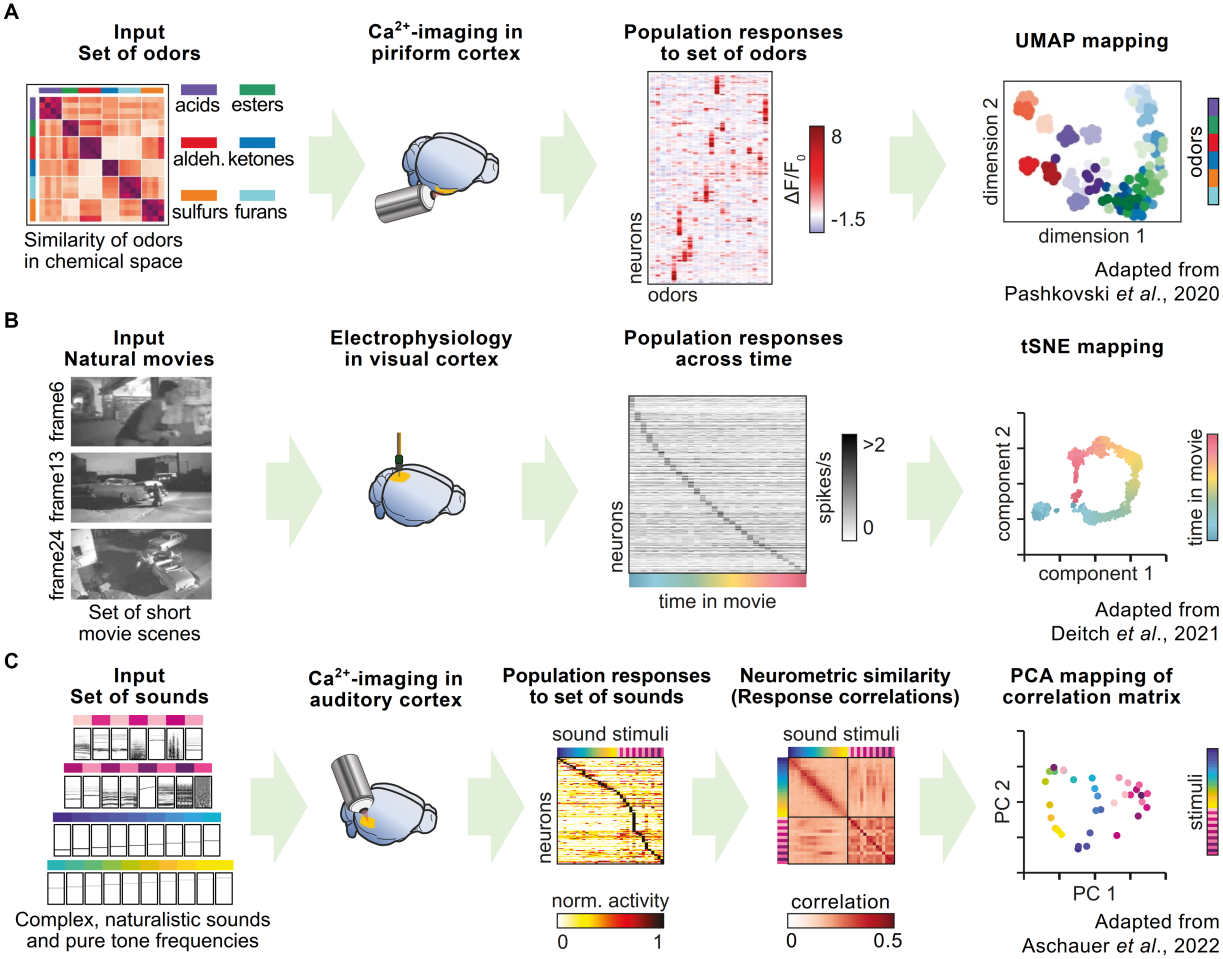
Representational maps in rodent neuroscience research. **(A)** Pashkovski et al. performed *in vivo* calcium imaging in piriform cortex and recorded the activity of local populations of neurons in response to a large set of odors, which could be chemically categorized into six different categories. UMAP embedding of neuronal response patterns shows a representational map reflecting the different odor categories. **(B)** Deitch et al. used a published dataset of Neuropixel recordings in the visual cortex during repeated presentation of short movie scenes. Individual neurons show selective firing to specific frames in the movie. tSNE mapping of population responses illustrate the temporal stimulus sequence as a representational map. **(C)** Aschauer et al. recorded neuronal activity in the auditory cortex using *in vivo* calcium imaging during the presentation of a large set of sounds, including pure tones and complex sounds. By combining the recordings from many individual mice, the authors established a global measure of representational similarity, which can be visualized with principal component analysis as a representational map.

Using *in vivo* calcium imaging of populations of neurons in the mouse piriform cortex, as well as the synaptic terminals of projection neurons stemming from the olfactory bulb, Pashkovski et al. (2020) showed that odor representations in the olfactory cortex, and its inputs from the olfactory bulb, are structured and organized by odor similarity. They simultaneously acquired response vectors of several hundred neurons after the presentation of a large odor set from different chemical categories. A correlation analysis showed that groups of neurons systematically represented the chemical relationships among the set of odors. By comparing different odors on a representational map estimated by UMAP (uniform manifold approximation and projection) embeddings, they could show that these relationships were conserved across different mice (Figure 3A). This representation could change with experience, demonstrating the flexibility of the olfactory cortex in updating odor representations.

Similarly, Deitch et al. probed neuronal population activity in visual areas of the mouse brain (Deitch et al., 2021) to show how representations change over time. They utilized two publicly available datasets of *in vivo* recordings (de Vries et al., 2020; Siegle et al., 2021) obtained from different recording techniques, namely Neuropixels probes (Jun et al., 2017), which are used for extracellular electrophysiological recordings, and calcium imaging of tens of thousands of neurons in six different brain areas of the mouse visual system. During the recordings, the mice were presented with an identical set of stimuli (short sequences of naturalistic movies). This dual approach has the advantage of compensating for potential biases introduced by the specific recording technique or by synthetic stimuli such as moving gratings, which are classically used in studies of the visual system. The authors showed that the stimulus representations in the visual cortex undergoes “representational drift,” meaning that the patterns of activity change over time in response to the visual stimulation (Chambers and Rumpel, 2017; Rule et al., 2019). These changes in representation can be influenced by experience and modulated by behavioral and attentional processes (Figure 3B).

The study of learning-induced changes of neural representations and their relation to the ongoing dynamics of sensory representations provide insight into the mechanisms underlying learning and adaptation, or attention and task engagement (Xin et al., 2019). In the auditory modality, Aschauer et al. used *in vivo* calcium imaging to study the long-term sensory representations of a large set of sound stimuli in the auditory cortex (Aschauer et al., 2022). The authors demonstrated that by combining the data from individual mice, it was possible to create a global representational map of stimulus similarity, which accounts for the perceptual behavior of individual mice (Figure 3C). The global similarity between activity patterns evoked by auditory stimuli predicted the level of stimulus generalization after fear learning, i.e., the ability of an individual to respond to an unconditioned stimulus in a similar way as to the conditioned stimulus. Behavioral generalization is considered not to reflect the limits of perception, but rather as a valid strategy in learning, given that the exact same stimulus is rarely encountered twice in real life. Using the same dataset, another study showed that despite the large drift in single neuron responsiveness, the tonotopic map – the classical functional organization of many auditory areas in the brain – is stably maintained (Chambers et al., 2022).

These exemplary studies highlight how the concept of representational maps has been successfully translated from human and non-human primate research to the rodent, a widely used model of choice in systems neuroscience.

## Experimental estimation of a representational map

The assessment of representational maps from large-scale neural data offers a general approach to estimate neural representations and their relationships to each other. In the following section, we will discuss practical considerations when estimating a representational map from neural recordings.

### Conceptualization

As a first step, the neural representations to be assessed should be clearly conceptualized and circumscribed with respect to sensory stimuli used and their ethological significance, the brain area and context of recorded activity. Different representations probed in an experiment do not, though, need to be unimodal. For example, one can present visual and auditory stimuli while recording neural activity in an auditory brain region to estimate a multisensory representational map. However, combining estimates of irrelevant or conflicting representations could make it difficult to interpret the resulting representational map.

### Size of the neural measurement

The neural measurement used to assess a representational map should have an adequate size. First, a sufficient number of representational elements (e.g., different sensory stimuli) should be probed experimentally. A wide range of stimuli presented in an experiment [e.g., at least several tens as in Kriegeskorte et al. (2008b) and Freiwald and Tsao (2010)] sets the basis for a reliable and precise representational map.

Besides the number of assessed representations, the number of recorded neural units (e.g., neurons, local fields, or fMRI voxels) should also be sufficiently high, as they determine the maximal dimensionality of a representational map and consequently the precision of the relations between mapped elements. Here, by way of example, representative studies have estimated representational maps based on population sizes in the range of hundreds (see, e.g., Kriegeskorte et al., 2008b) up to several tens of thousands of neuronal units (see, e.g., Aschauer et al., 2022). Subsampling the dataset offers a practical approach to assess the robustness of the map estimate. Ideally enough data can be obtained from a single individual to obtain a sufficiently representative sampling of neuronal population activity. Technical limitations, however, often require pooling data from multiple individuals, leading to an estimate of a representational map that shows common features. Datasets from two pooled, but independent datasets should converge on a comparable structure, when the sample size is representative enough.

### Types of data and algorithms to create the representational map

The basis for assessing a representational map is set by the underlying measure of neural activity. This measure is influenced by the method of probing activity (e.g., calcium imaging, electrophysiological recordings, or fMRI), and the metric of activity (e.g., continuous ΔF/F_0_ traces or binarized activity). The activity patterns of all recorded units over all presented sensory stimuli then constitute an estimation of their neural representations. The estimates of representational maps can be generated either from the activity patterns of the neurons themselves, measured in units such as action potentials or calcium events, or from the structure of the correlations between activity patterns. The latter case may be useful in cases where signal strength varies across measured units due to experimental factors rather than real variations in the underlying activity. RSA, for example, relies on a correlation analysis that normalizes the information of each neural unit, hence disregarding the overall magnitude of activity. In this way, fMRI voxels that may vary in their maximal signal strength can still be used reliably to assess a representational map. Furthermore, correlation-based approaches were successfully applied to represent aspects of stimulus discrimination (Kriegeskorte et al., 2008b; Hiramatsu et al., 2011; Bathellier et al., 2012; Connolly et al., 2012; Groen et al., 2012; Aschauer et al., 2022).

Recordings of neural activity that provide a larger degree of homogeneity between neural units (e.g., calcium imaging with single-cell resolution) can be directly used to estimate a representational map by reducing its dimensionality without prior construction of a similarity matrix. For this direct estimation of a representational map multiple approaches, such as principal component analysis, are available. Covariance-based estimations of representational maps can maintain information about response magnitudes and hence include aspects of stimulus salience (Treue, 2003; Ceballo et al., 2019).

Accordingly, the distance metrics used for estimating a representational map should also be chosen based on a hypothesis of the main feature in neural population activity that reflects stimulus relationships. Commonly used distance metrics are dissimilarity 1-linear or rank-based correlation coefficient (Kriegeskorte et al., 2008a), the Euclidean distance between population activity vectors or the absolute activity difference (Kriegeskorte et al., 2008a; Walther et al., 2016). Additionally, if the distributional distance between representational entities is of interest, the Mahalanobis distance can be used (Kriegeskorte et al., 2006; Kriegeskorte and Kievit, 2013). Since RSA is based on the assumption that there is a categorical structure in a dataset of neural activity, clustering methods can be helpful to reveal such categorical divisions. While unsupervised, hierarchical clustering (Laakso and Cottrell, 2000; Kriegeskorte et al., 2008b; Nili et al., 2014; Kaneshiro et al., 2015) has been frequently applied, a supervised approach could improve and emphasize behaviorally relevant categorization (Khaligh-Razavi and Kriegeskorte, 2014).

### Measures of trial-to-trial variability

In many cases, the similarity matrices used in a RSA are constructed from trial-averaged population activity vectors (Kriegeskorte et al., 2008a), hence neglecting inter-trial variability in neuronal activity. However, the correlation in trial-to-trial variability of single neural units, or noise correlation, carries relevant information for population codes (Averbeck et al., 2006; Kohn et al., 2016; Panzeri et al., 2022). To capture this information, an alternative way of constructing representational similarity matrices utilizes crosswise correlations of single trial estimates of representations (Bathellier et al., 2012; Aschauer et al., 2022; Filipchuk et al., 2022). Here, the correlation across combinations of single trials is averaged for each pair of probed representations, yielding a representational similarity matrix in which the diagonal (self-correlation) does not equal 1 by construction. This approach has the advantage of providing an additional measure of trial-to-trial reliability for each assessment of a representation, reflected by its mean self-correlations.

### Brain states and behavioral context

Neural computations can be affected by global brain states (Zagha and McCormick, 2014; McGinley et al., 2015; Li et al., 2019; Bradley et al., 2022) that likely also affect the estimate of representational maps (Cauda et al., 2012; Filipchuk et al., 2022). In this respect, different brain states could, for instance, suppress elements on a representational map or change their position. Therefore, experiments should be controlled for such state-dependency, either by standardizing the conditions of an experiment, leading to more comparable brain states, or by measuring brain state explicitly (e.g., with pupillometry or measurement of large-scale brain oscillations) to clarify the specific effects of different brain states on representational maps.

Behavioral context, e.g., involving a subject performing a task versus measuring responses to the same stimuli in a passive setting can also have substantial effects on neuronal activities (Otazu et al., 2009) and therefore also affects the estimates of representational maps derived from them. This may apply particularly for representational maps in hierarchically higher brain areas increasingly incorporating contextual information on sensory stimuli that is largely determined by the task setting. However, the application of sensory stimuli in a defined task setting may also give rise to specific expectations in an individual that can impact on population responses in sensory early areas (Keller and Mrsic-Flogel, 2018).

### Validation of a representational map

To ensure that the relationships of the elements on a representational map are meaningful in respect to neural computation, they need to be validated. This can be accomplished by comparing the relational structure of a representational map with the structure of the stimulus set used to probe the mapped representations. Here, stimuli can be related to each other with respect to their physical properties (e.g., tone frequency and intensity) or experimental conditions (e.g., paired presentation in an experiment), allowing *a priori* hypotheses about a map estimated from these representations.

In addition, a quantitative validation can be achieved by correlating the distances between the probed elements on a representational map with a readout of behaviors, psychophysics and other exophenotypes related to the respective represented entities. Here, multiple studies have demonstrated a link between representational maps and behaviorally relevant categorization of represented entities (Battaglia et al., 2004; Bathellier et al., 2012; Maurer et al., 2014; Aschauer et al., 2022). Technical developments in the future may enable the manipulation of neural activity in order to shift elements on a representational map and affect associated behavior. Such manipulation experiments require a detailed understanding of representational maps and the underlying neural codes, but are critical for the assertion that a representational map reflects meaningful information and causally determines behavior.

## Collective statistics of the tuning of individual neurons determine the structure of a representational map

Understanding the link between the activity of individual neurons that collectively form a representational map can provide interesting constraints and insights on the activity patterns emerging in neuronal circuits. Before the advent of large-scale, single cell recording techniques, neuroscientists typically described sensory-evoked neuronal activity in the form of tuning curves, often obtained from a comparably small number of neurons. In this section, we tasked ourselves with assembling a large-scale dataset by picking individual tuning curves that together would form the basis of a well-structured representational map. Would our choices really matter, or would any assembly of tuning curves result in some form of representational map?

As mentioned at the beginning of this review, if all representational entities were associated with activity patterns that are equally dissimilar from each other, no meaningful representational map could emerge. At the level of individual cells, this hypothetical scenario could correspond to a situation in which only one specific neuron would fire for each representational entity on the map. This extreme example shows that not all combinations of tuning curves can serve as a basis for a representational map and some redundancy in tuning of individual neurons appears to be essential. Then, what are the statistics of tuning curves in the brain that collectively can form the basis of a meaningful representational map?

**Figure 4:**
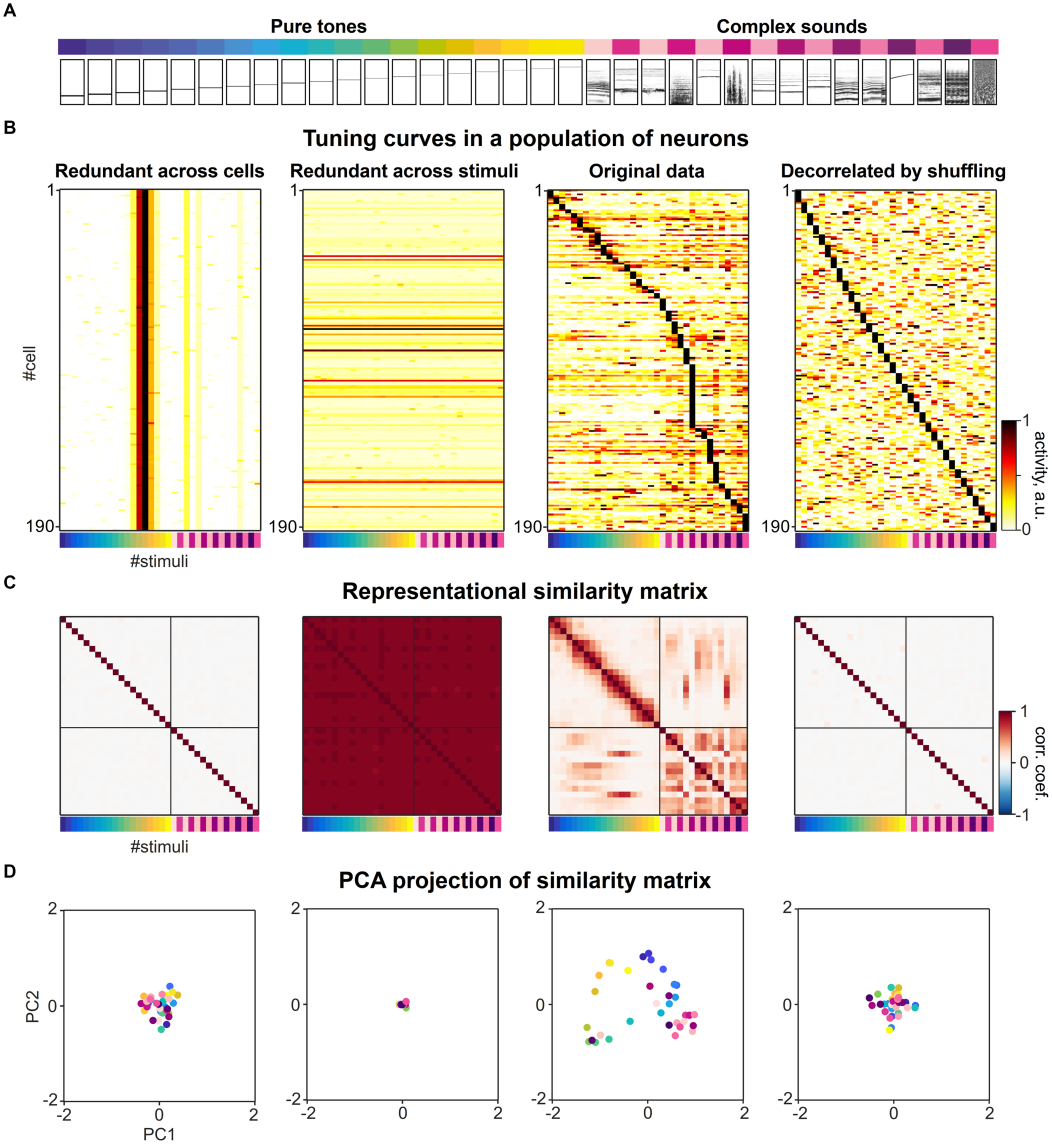
The collective statistics of the tuning curves of individual neurons shapes the structure of a representational map. **(A)** A set of sounds (19 pure tones ranging between 2 and 45 kHz and 15 complex sounds) presented in the previous study (Figure 3C, Aschauer et al., 2022). The relational structure between these sounds is investigated in neuronal activity space. **(B)** Sound-evoked tuning curves across different sound stimuli in individual single neurons, adapted from Aschauer et al. (2022). Second from right: original experimental data. Left: Artificial population of neurons with extreme redundancy in tuning curves across neurons, simulated by replicating a tuning curve of a single neuron from the experimental data and adding noise. Second from left: Artificial population of neurons with extreme redundancy across population responses for different stimuli, simulated by replicating one specific population response vector for a stimulus from the experimental data and adding noise. Right: Artificial population of neurons with highly diversified tuning curves constructed by randomly shuffling the real dataset across cells and stimuli. The matrix was sorted by maximal stimulus response for each neuron. The colormap represents normalized evoked neuronal activity in arbitrary units. **(C)** Representational similarity matrices constructed from the datasets in A. The Pearson correlation of the two population response vectors to a given pair of sound stimuli was calculated as metric of similarity. The colormap represents Pearson correlation coefficients of population response vectors evoked by the set of 34 stimuli. **(D)** Dimension-reduced display of the representational map. Principal component analysis was applied to the representational similarity matrices in C and the first two principal component scores of each correlation pattern to each sound stimuli were mapped onto the corresponding eigenvectors. The color of each dot represents stimulus identity as in A, B and C.

We considered a previously published dataset from various subfields of the mouse auditory cortex consisting of responses to a diverse set of 34 different sound stimuli (Figure 4A) recorded in 21,506 neurons (Aschauer et al., 2022). When sorting the response of the neurons according to their maximal response, a diverse range of tuning curves tile the space defined by the set of sound stimuli. The tuning of individual neurons is relatively narrow and typically only a few of the 34 stimuli evoke a significant response in a given responsive neuron (Figure 4B, second from right). To understand the impact of tuning curve diversity on the formation of a representational map, we created artificial datasets in which (i) all neurons were assigned the same tuning as one of the experimental cells (Figure 4B, left), or (ii) all population response vectors were assigned the same response pattern for all stimuli (Figure 4B, second from left), respectively. In both artificial conditions, uniform noise was added to the response patterns. While the former artificial dataset has extreme redundancy in tuning across cells, the latter dataset is diverse in tuning, but extremely redundant in population response vectors across stimuli.

In order to create an artificial dataset in which the redundancy of tuning curves across neurons and the redundancy across population response vectors for different stimuli is much less than for the experimental data, we shuffled the response matrix vertically and horizontally, randomly distributing the 34 response patterns over neurons (Figure 4B, right). This artificial dataset represents a decorrelation of neuronal responses at a much higher level than experimentally observed and thereby would allow a decoding of stimuli that is even more effective compared to the original recordings.

We constructed representational similarity matrices from the four datasets in which the Pearson correlation was calculated as a measure of similarity for all pairwise population responses (Figure 4C). As an illustration of the structure of the resulting similarity matrix, we performed a principal component analysis and plotted the individual stimuli along the first two principal components of the similarity matrix (Figure 4D). When considering the off-diagonal entries of the similarity matrix from the original dataset, distinct patterns of higher and lower pairwise correlations were found across subsets of stimuli, that characterize the structure of the representational map (Figures 4C,D, second from right). In contrast, the representational similarity matrices of the population responses with extremely high levels of redundancy in tuning (Figure 4C, first and from left), show very little structure in the pairwise correlations across activity patterns, either on an overall low level (Figure 4C, first from left) or high level (Figure 4C, second from left). When comparing the similarity matrix obtained from the original data with the matrix constructed from shuffled data representing an extremely diversified tuning of individual neurons, we also observed a loss of structure in the pairwise correlations across activity patterns (Figure 4C, second from right).

Taken together, these simulations of highly redundant or highly diverse sets of neural responses present two extremes that set the boundaries in which the brain organizes its activity. The shape and width of individual tuning curves and the redundancy in neurons with similar tuning may not reflect the maximal possible decorrelation of response patterns to various sensory stimuli that could be implemented in brain circuits, but instead are essential properties that mediate the encoding of relationships between representational entities and thereby determine the structure of representational maps (Stringer et al., 2019). The encoding of similarity by correlated tuning curves also explains why representational maps must occupy only a lower-dimensional subspace within the full space of theoretically available neuronal activities. Furthermore, we hypothesize that network mechanisms are likely operating to safeguard an optimal balance between the decorrelation and correlation of sensory evoked activity patterns and allow a representational map to emerge.

## Maintenance of representational maps and their adaptive and maladaptive plasticity

Representational maps reflect how the brain encodes and represents different representational entities in a relational context, and are thought to underlie computational processes carried out with the represented information. Here, a well-balanced interplay of stability on the one side and plasticity on the other ensures that a representational map can be adjusted to changing environmental conditions, while at the same time maintaining essential relational properties. Thus, new information can be embedded into preexisting knowledge without corrupting previously stored information. In the following section, we elaborate on aspects of the plasticity and stability of representational maps, and demonstrate how they can be linked to clinical conditions.

### Maintaining and adjusting representational maps

It has been shown that large scale topographic maps in the brain – special forms of representational maps, e.g., the tonotopic map of sound frequency – can be reliably obtained from recordings of cortical activity over time (Guo et al., 2012), indicating a general level of stability. However, during altered environmental conditions or in the context of learning, neural representations have been shown to change and reorganize substantially, affecting their neural code and mapping (Merzenich et al., 1984; Clark et al., 1988; Pons et al., 1991; Kilgard and Merzenich, 1998). By way of example, sensory deprivation leads to a remodeling of functional cortical maps, where deprived cortical areas are taken over by other sensory systems that are used for compensation (Pons et al., 1991; Finney et al., 2001; Elbert and Rockstroh, 2004; Weiss et al., 2004). This illustrates that the intrinsic plasticity of representational maps in the brain reflects the changes in stimulus statistics by rearranging, expanding or adding represented elements (Amedi et al., 2003; Hamilton et al., 2004; Thaler et al., 2011; Behrens et al., 2018; Freund et al., 2021).

The apparent stability of a representational map under stable sensory and environmental conditions, however, arises from a dynamic equilibrium of the individual neuronal responses that collectively encode the map at a global level. It has been shown that sensory responses in individual neurons can exhibit substantial intrinsic volatility over the time course of few days, even under basal conditions without reinforced environmental cues or other learning tasks (Driscoll et al., 2017; Rule et al., 2020; Schoonover et al., 2021; Aschauer et al., 2022). This raises the central question: How can a representational map maintain stable relations between the representational entities if the functional properties of the neurons encoding these elements are intrinsically volatile? Several solutions to this problem have been discussed, including the following two proposals: First, drift could occur in a coordinated manner for the various representational entities on a map in such a way that the elements changing their location on the map would preserve their distance and relation to each other (Xia et al., 2021). Second, intrinsic drift could affect only a subpopulation of the neurons that form a representational map which do not strongly affect relations of mapped elements. From this perspective, representational drift could affect neurons to a variable degree (Chambers and Rumpel, 2017; Pettit et al., 2022), primarily affecting those neural populations that encode information which is redundant or orthogonal to the space of a representational map. Here, recent experimental evidence indicates that the neurons forming a representational map consist of a drifting subpopulation serving the generation of a local topographically organized sensory response, in a balanced manner over time and space (Chambers et al., 2022). Thus, the overall structure of the map can be preserved, as long as the collective statistics of the tuning curves in the subpopulation of responsive neurons are maintained.

Together, these considerations underline the role of tuning width and redundancy in tuning curves in the neural code defining a map. By regulating these parameters, different neural mechanisms can regulate the plasticity and stability of a representational map in order to create a computational readout that is largely flexible, but also maintains prior knowledge in the form of relational distances between representational entities.

### Clinical implications

**Figure 5:**
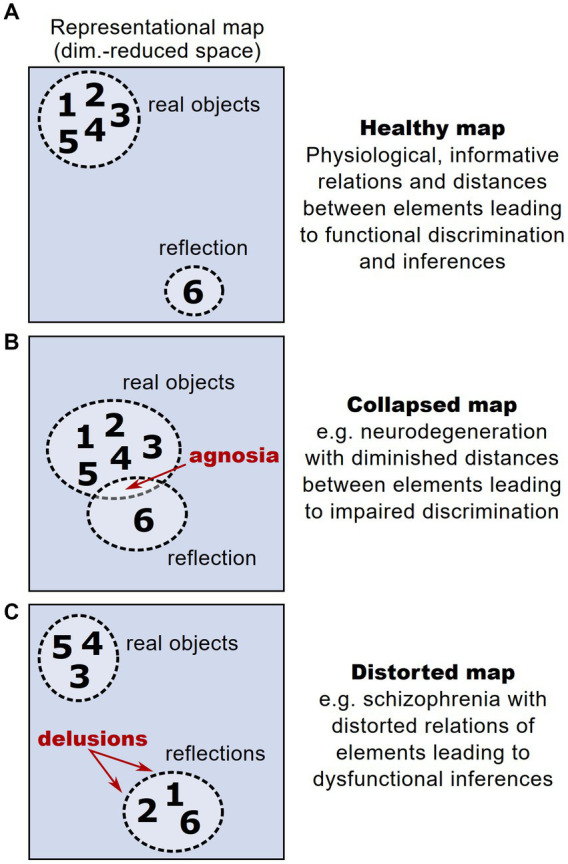
Altered representational maps and their clinical implications. **(A)** Schematic higher-order representational map under healthy conditions, taken from the example in Figure 1. Each number reflects a given representational entity. In this example, the map allows a discrimination of entities according to a cluster of real objects vs. reflected objects. **(B)** Analogous schematic of a representational map under the condition of a drop-out of neural units, e.g., in neurodegeneration. Here, due to the loss of neural dimensions, the maximal distance between represented entities is reduced, impeding their discrimination and potentially leading to agnosia. **(C)** Analogous schematic of a representational map under the condition of aberrant neural activity patterns during psychosis, leading to distorted relations between represented entities. The distortions can lead to misattribution of some entities to a wrong category, potentially leading to symptoms like delusions.

The interplay between stability and plasticity is fundamental for the functionality of representational maps as valid and operational models of an organism’s knowledge. In contrast to physiological conditions, where a representational map faithfully encodes relationships between different representational entities, thereby enabling valid inferences (Figure 5A), aberrant maintenance or plasticity of representational maps can lead to distorted perceptual experience. This constitutes a novel perspective on different psychopathologies and their symptoms. For example, the problem of maintaining functional representational maps in the face of a progressive drop-out of neural units is particularly relevant in neurodegenerative disorders. In these disorders, the loss of neurons encoding representational entities could dramatically affect and distort the structure of a representational map. However, patients with neurodegenerative disorders and pronounced loss of neurons typically show a significant period without any or at least with very mild cognitive impairments (Villemagne et al., 2013; Aisen et al., 2022), hinting that compensatory mechanisms safeguard the structure of a representational map (Ebrahimi et al., 2022). After this latent period, early clinical symptoms typically affect the cognitive ability of discriminating and recognizing different objects (termed agnosia), setting them in a meaningful context and extracting knowledge from them (Kocagoncu et al., 2021; Tran et al., 2021). Thus, the onset of clinical symptoms could be interpreted as the moment in which a representational map’s structure starts to deteriorate: While initial degeneration of neurons hardly affects a representational map’s structure, the broad loss of neurons reduces the dimensionality and hence the overall discriminability of representational entities, leading to agnosia (Figure 5B).

Furthermore, representational maps are thought to reflect the structure of perception and consecutive decision-making (Schuck et al., 2016; Freund et al., 2021). This implies that distortions of a representational map comprising perceptual entities can cause perceptual impairments such as delusions and hallucinations (Figure 5C). Compelling clinical examples for these perceptual deficits are found in psychotic disorders such as schizophrenia, which is typically associated with impaired cognitive abilities and sensory integration (Carter et al., 2017). Strikingly, schizophrenic patients have been found to show altered and dysfunctional cognitive map architectures (Nour et al., 2021). These observations only recently gave rise to the hypothesis that distortions of representational maps arise from deficits in maintaining stable brain states that are necessary to form functional representational maps (Braun et al., 2021; Musa et al., 2022).

Distorted representational maps could also offer an explanation for maladaptive learning and dysfunctional associations, for instance in the context of addiction or anxiety disorders. Here, dysfunctional associations are thought to play a central role in maintaining and consolidating psychopathology (Luscher and Malenka, 2011; McKim and Boettiger, 2015; Ball and Gunaydin, 2022), e.g., when a drug becomes associated with common environmental cues, leading to increased relapse probability, or when vastly innocuous actions, such as walking over a busy street, become linked with extensive fear leading to avoidance and social isolation. In these scenarios, representational maps could offer an impartial estimation of individual burden, but also of treatment success during a therapy, indicated by loosening the dysfunctional pairing on the map (Beckers et al., 2023). Moreover, individual representational maps could be used as a complementary diagnostic tool, reflecting individually learned associations and concepts, which might predispose subjects to develop particular disorders.

Hence, one promising focus of future research lies in standardizing the conditions for assessing representational maps, in order to quantitatively compare healthy and mentally ill individuals. As the concept of representational maps implies that psychopathological conditions could be reversed if one could identify and correct the aberrant tuning patterns of neural units, different therapeutic interventions could be tested in order to restore physiological conditions of a representational map. Here, treatment approaches that show a broad effect on different cognitive levels seem promising: For instance, psychedelic substances have recently been shown to induce destabilized states in cognitive models (Ballentine et al., 2022), increase neuroplasticity (de Vos et al., 2021) and enhance cognitive flexibility (Doss et al., 2021; Mason et al., 2021). These substances are therefore interesting candidates to manipulate neural representations. In general, such treatments should aim to selectively destabilize a representational map and hence make it susceptible to learning-related remodeling [e.g., in the context of psychotherapy (Musa et al., 2022)] in order to restore physiological conditions.

## Conclusion and outlook

In this review, we conceptualized representational maps, in which not only the identities of representational entities are encoded as distinguishable neuronal activity patterns, but also their relationships are encoded as similarity in a higher-dimensional space of neural activity. We highlighted several studies and their methodologies to experimentally assess the structure of representational maps and discussed how they can emerge from individual neurons with diverse tuning properties. Furthermore, we showed how symptoms of brain diseases can be interpreted in the framework of representational maps. We believe that in the future this framework will enable versatile descriptions of the organization of neural representations in basic and clinical research, possibly guiding the development of methodologies allowing specific manipulations of these maps in an experimental and therapeutical context. Although we have focused our review on the structure of representations in biological networks, the concept of representational maps appears to be a more general principle that could be also instrumental in gaining insight in the structure of artificial neuronal networks (Kriegeskorte, 2015; Dwivedi et al., 2021; Saxe et al., 2021).

## Author contributions

TN: Writing – original draft, Writing – review & editing. DA: Writing – original draft, Writing – review & editing. AC: Writing – original draft, Writing – review & editing. JS: Writing – original draft, Writing – review & editing. SR: Conceptualization, Writing – original draft, Writing – review & editing.
